# Effects of Occupational Exposure to Waste Anesthetic Gas on Oxidative Stress and DNA Damage

**DOI:** 10.1155/2021/8831535

**Published:** 2021-01-19

**Authors:** Hai-Xin Hua, Hai-Bo Deng, Xiu-Ling Huang, Chang-Qing Ma, Ping Xu, Ye-Hua Cai, Hai-Tang Wang

**Affiliations:** ^1^Department of Anesthesiology, Zhujiang Hospital of Southern Medical University, 253 Gongye Ave., Guangzhou, China 510282; ^2^Department of Anesthesiology, Huizhou Third People's Hospital, Guangzhou Medical University, No. 1, Xuebei Street, Huicheng District, Huizhou City, Guangdong Province, China; ^3^Department of Anesthesiology, Nanfang Hospital of Southern Medical University, 1838 Guangzhoudadaobei, Guangzhou, China 510515

## Abstract

**Objective:**

The aim of the study was to investigate the potential effects of waste anesthetic gas (WAG) on oxidative stress, DNA damage, and vital organs.

**Methods:**

A total of 150 members of the staff at a hospital were assigned to an exposure group or control group. The 68 operating room (OR) staff in the exposure group were exposed to WAG, and the 82 non-OR staff in the control group were not exposed to WAG. Air samples were collected in the OR, and the sevoflurane concentrations in the samples were determined. Superoxide dismutase (SOD), glutathione peroxidase (GSH-px), and malondialdehyde (MDA) in plasma from the participants were determined to assess oxidative stress. Western blot analysis was used to detect *γ*H_2_AX in peripheral blood to assess DNA damage. Hematopoietic parameters, liver function, kidney function, and changes in electrophysiology were assessed to identify the effects on the vital organs.

**Results:**

The mean (±standard deviation) sevoflurane concentration in 172 air samples from 22 operating rooms was 1.11 ± 0.65 ppm. The superoxide dismutase activity and vital organ parameters (lymphocyte, hemoglobin, and total protein concentrations and heart rate) were significantly lower (*P* < 0.05) in the exposed group than the control group. The malondialdehyde, total bilirubin, and creatinine concentrations and QT and QTc intervals were significantly higher (*P* < 0.05) in the exposed group than the control group. There were no significant differences between the glutathione peroxidase activities and *γ*H2AX concentrations for the exposed and control groups.

**Conclusions:**

Long-term occupational exposure to waste anesthetic gas may affect the antioxidant defense system and probably affects vital organ functions to some extent. No correlation between DNA damage and chronic exposure to WAG was observed.

## 1. Introduction

Inhaled anesthetics are widely used because the dose can be readily controlled. However, waste anesthetic gas (WAG) can leak into an operating room (OR). Medical personnel in ORs suffer long-term exposure to WAG [1]. There is concern about the risks posed to the health of people suffering long-term exposure to WAG. It has been found that occupational exposure to WAG can cause an imbalance between oxidation and antioxidation, changes in antioxidant enzymes, increases in oxygen free radical concentrations, DNA damage, and even genotoxicity [2–5]. Large epidemiological investigations have indicated that exposure to WAG can increase the risks of chronic diseases (e.g., liver dysfunction and renal insufficiency), spontaneous abortion, and congenital malformation occurring and can decrease the birth rate and increase the stillbirth rate [6–8]. However, most of these studies were performed decades ago, and few relevant studies have been published in recent years. There have recently been great changes in anesthesiological methods, drugs, and equipment. Sevoflurane and desflurane have gradually replaced nitrous oxide, enflurane, and isoflurane and are now widely used in clinical anesthesiological practice. New ventilation strategies, such as low tidal volume ventilation, that are increasingly used by clinical anesthesiologists may decrease WAG emissions. Laminar flow systems and scavenging systems, which are now widely used in ORs, may decrease WAG emissions. These changes may have decreased WAG concentrations in ORs and the effects of WAG exposure on OR staff [9]. The effects of WAGs on medical staff in ORs need to be reassessed. In this study, WAG concentrations in ORs were investigated, and potential effects of occupational exposure to low concentrations of sevoflurane in OR staff were assessed by investigating oxidative stress, DNA damage, changes in hematopoietic parameters, liver function, kidney function, and electrophysiological changes.

## 2. Materials and Methods

### 2.1. Study Design and Participants

The study was approved (approval no. 2017-MZK-001) by the Medical Ethics Committee of Zhujiang Hospital, part of the Southern Medical University, and was registered in the Chinese Clinical Trial Registry (registration no. ChiCTR-IOR-17013915). The cross-sectional study was performed at Zhujiang Hospital, part of the Southern Medical University, Guangzhou, China. Written informed consent was obtained from each participant. The participants, full-time Zhujiang Hospital staff aged 22–50 y with 2–25 y service, were assigned to two groups. The exposure group contained OR staff who were exposed to anesthetic gases but not to any other hazardous agents. The control group contained non-OR staff matched to the exposure group in terms of age, sex, length of service, smoking habits, and drinking habits and with no occupational exposure to hazardous agents such as radiation and anesthetic gases. The exclusion criteria were (1) history of anesthesia, surgery, radiological diagnosis, or treatment in the previous three months; (2) current pregnancy or lactation; (3) autoimmune disease, malignancy, infectious disease, or other acute or chronic disease that could affect oxidative stress and DNA levels; (4) long-term use of vitamin supplements or antioxidants; and (5) refusal to participate in the study.

### 2.2. Sevoflurane Concentration in the OR

The Zhujiang Hospital ORs used for the study were equipped with air conditioning systems and scavenging devices. Sevoflurane has been the only available inhaled anesthetic for several decades and is used frequently to induce and maintain anesthesia. Air samples were collected from different sites within the ORs between 8:00 and 8:30 a.m. on weekdays. Anesthetists and anesthetic nurses each wore a sampler close to (~10 cm from) the respiratory area during personal exposure (P1). Each sampler was turned on as soon as sevoflurane started to be administered. Each sampler had a flow rate of 100 mL/min, and the sampling tube was replaced every 2 h. Sampling continued for 8 h. Samplers were also placed on the nurses' tables in the ORs (P2). Each sampling tube was sealed immediately after the sample collection period had ended and then sent to the laboratory, where the sevoflurane concentration was determined using an HP6890N gas chromatograph (Agilent Technologies, Santa Clara, CA, USA).

### 2.3. Demographic Data

Demographic data for each participant (including age, sex, body mass index, length of service, occupation, smoking habits, and drinking habits) and clinical characteristics (including medical history and current medication) were recorded. Blood samples were collected from all of the participants and were preserved.

### 2.4. Oxidative Stress

Peripheral venous blood (2 mL) was obtained from each participant into a sterile tube containing heparin (an anticoagulant). The blood was centrifuged at 3500 revolutions/min for 10 min; then, the supernatant was transferred to a fresh tube and stored at −20°C until analysis. Each sampling tube was given a unique label, stored in a portable refrigerator, and transferred to the laboratory for processing within 3 h of collection. The investigators did not know which participant was allocated to which group. The superoxide dismutase (SOD) activity, glutathione peroxidase (GSH-px) activity, and malondialdehyde (MDA) concentration in each plasma sample were determined to allow oxidative stress to be assessed. The SOD activity, GSH-px activity, and MDA concentration were prepared using a superoxide dismutase assay kit (Beyotime Biotechnology, Shanghai, China), a lipid oxidation assay kit (Beyotime Biotechnology), and a glutathione peroxidase assay kit (Nanjing Jiancheng Bioengineering Institute, Nanjing, China), respectively, and the prepared samples were analyzed using a DR5000 ultraviolet/visible spectrophotometer.

### 2.5. DNA Damage and Vital Organ Functions

Peripheral venous blood (2 mL) was obtained from each participant into a sterile tube containing heparin and then diluted to 4 mL. The sample was then slowly added to a lymphocyte separation solution in a 20 mL centrifuge tube. The mixture was then centrifuged at 1500 revolutions/min for 20 min. The liquid separated into three layers. The upper layer was light yellow plasma, the middle layer was milky white and contained the white blood cells, and the bottom layer contained the blood cells. The white blood cell layer was collected using a pipette and placed in a sterile 1.5 mL centrifuge tube. The liquid was then centrifuged at 10000 revolutions/min for 5 min, and the white precipitate was transferred to another tube and stored at −80°C for Western blot analysis. Additional DNA damage was avoided by performing every step under indirect light. *γ*-H2AX protein in each sample was measured using a Western blot analysis kit (Promega, Madison, WI, USA) to assess DNA damage.

Peripheral venous blood (2 mL) was obtained from each participant into a tube containing EDTA (an anticoagulant) to allow a complete blood count to be performed, and 4 mL of venous blood was obtained into a dry tube without additives for use in biochemical tests to assess blood lipid concentrations, liver function, and renal function. Each tube was labeled and then analyzed. Complete blood counts were performed using an SE5000 automatic blood cell analyzer. Blood biochemical analyses were performed on the day the blood samples were collected using a Mindray BS2001 automatic biochemical analyzer. A 12-lead electrocardiogram (ECG) of each subject in a resting state was acquired in the morning using an ECG-9620P2 ECG machine (Shanghai). The same technician acquired the ECGs of all the participants. The ECG results were analyzed by another technician who did not know to which group each participant had been allocated.

### 2.6. Sample Size Calculation

The sample size was calculated using Formula 1, which was published previously [10]. The difference (*D*), standard deviation (*S*), and test efficiency were 3.3, 5.4, and 8, respectively. The ratio between the participants in the exposure and control groups was 1. We estimated that 172 participants (86 in each group) were required.

(1) $$\begin{array}{c} N=\frac{2MS^{2}}{D^{2}}, \end{array}$$

Formula 1 is used to determine the sample size (*N*). *M* is the test efficiency, *S* is the standard deviation, and *D* is difference.

### 2.7. Statistical Methods

Data analysis was performed using the SPSS 20.0 software (IBM, Armonk, NY, USA). If a dataset had a normal distribution, the data were represented as $\bar{x}\pm s$. Independent sample *t*-tests were used to identify differences between two independent samples. The median (lower quartile, upper quartile) were used to describe data that did not follow the normal distribution. Nonparametric rank-sum tests were used to compare groups of data. Chi-square tests or Fisher's exact probability tests were used to analyze enumeration data. The alpha level was 0.05 (bilateral). *P* < 0.05 was considered to indicate a statistically significant result.

## 3. Results

### 3.1. Sevoflurane Concentrations in the ORs

**Figure 1:**
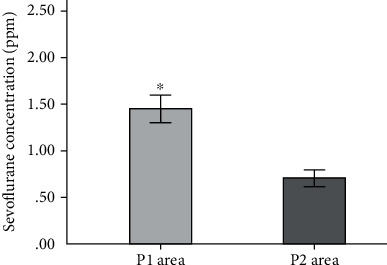
Sevoflurane concentrations in the different working areas. Notes. P1: anesthesiologist and anesthesia nurse working areas; P2: operating room nurse working areas.

A total of 172 air samples from 22 ORs were collected. The sevoflurane concentration in each sample was determined. A total of 88 of the samples were collected in areas in which anesthesiologists or anesthesia nurses worked (P1), and 84 samples were collected from areas in which OR nurses worked (P2). The sevoflurane concentrations were 0.07–3.84 ppm (mean 1.11 ± 0.65 ppm). The sevoflurane concentrations in the P1 samples were 0.26–3.84 ppm (mean 1.44 ± 0.65 ppm). The sevoflurane concentrations in 28% of the P1 air samples exceeded the Chinese standard for occupational exposure. The sevoflurane concentrations in the P2 samples were 0.07–1.78 ppm (mean 0.70 ± 0.36 ppm). The sevoflurane concentrations in >8% of the P2 air samples exceeded the Chinese standard for occupational exposure. The sevoflurane concentrations were significantly higher in the P1 samples than the P2 samples. The results are shown in Figure 1.

### 3.2. Demographic Characteristics

A total of 180 members of staff were screened for eligibility, and 153 met the inclusion criteria. A total of 150 members of staff were included in the study (one was excluded because of pregnancy, one because of a chronic infectious disease, and one because the serum creatinine concentration was above the upper limit of the normal range). Of the 150 participants, 68 were OR personnel (anesthesiologists, anesthesia nurses, itinerant nurses, and instrument nurses) and were assigned to the exposure group. The control group contained 82 non-OR staff from the departments of digestive medicine, hematology, rehabilitation, rheumatic immunology, ultrasound, respiratory medicine, pediatrics, and geriatrics. There were no significant differences between the demographic characteristics of the two groups. The demographic characteristics of the groups are shown in Table 1.

### 3.3. Oxidative Stress

**Figure 2:**
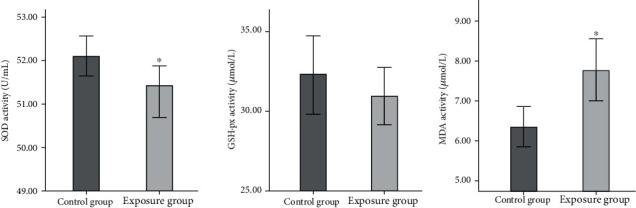
Superoxide dismutase (SOD) activities, glutathione peroxidase (GSH-px) activities, and malondialdehyde (MDA) concentrations for the exposure and control groups. Note. (1) Compared with the control group; (2) ^∗^*P* < 0.05.

Normal distribution tests were performed on the SOD activity, GSH-px activity, and MDA concentration data. The SOD activity data did not follow a normal distribution, so the median and quartiles were used in comparative analyses. The median (lower quartile, upper quartile) SOD activities were 51.42 (49.80, 52.27) U/mL for the exposure group and 52.11 (51.19, 52.80) U/mL for the control group. A nonparametric rank-sum test indicated that the SOD activities for the exposure and control groups were significantly different (*Z* = −2.84). The MDA concentration was higher for the exposure group (7.77 ± 2.99 *μ*mol/L) than the control group (6.35 ± 1.81 *μ*mol/L). The GSH-px activities for the exposure group (30.93 ± 6.19 *μ*mol/L) and control group (32.33 ± 7.54 *μ*mol/L) were not significantly different (Figure 2).

### 3.4. DNA Damage

**Figure 3:**
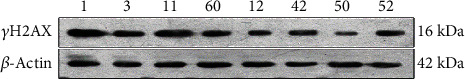
*γ*H2AX and *β*-actin bands for the exposure and control groups. Notes. The exposure group samples contained the 1, 3, 60, and 42 bands, and the control group contained the 11, 12, 50, and 52 bands

**Figure 4:**
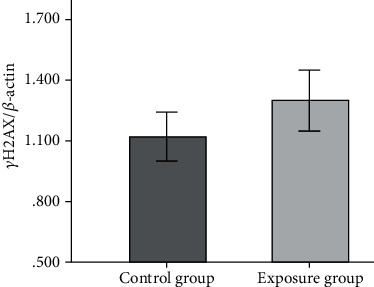
*γ*H2AX/*β*-actin ratios for the control and exposure groups. No difference was found between the *γ*H2AX expression in the control and exposure groups.

Western blot analysis was performed to detect the *γ*H2AX expression in the exposure and control group samples. The *γ*H2AX and *β*-actin bands for the two groups are shown in Figure 3. The *γ*H2AX/*β*-actin ratios for the exposure and control groups were 1.29 ± 0.51 and 1.12 ± 0.42, respectively. The *γ*H2AX expression results for the exposure and control groups were not significantly different. The results are shown in Figure 4.

### 3.5. Complete Blood Count

The hemoglobin concentrations in the exposure and control group samples were 121.75 ± 12.89 and 133.62 ± 16.56 g/L, respectively. The hemoglobin concentrations were much lower in the exposure group samples than the control group samples. The lymphocyte concentrations were lower in the exposure group samples (2.31 ± 0.51 × 10^9^/L) than the control group samples (2.63 ± 0.82 × 10^9^/L). The white blood cell, blood platelet, red blood cell, neutrophil, eosinophil, and basophil cell counts for the control and exposure groups were not significantly different. The results are shown in Table 2.

### 3.6. Liver Function and Renal Function

The total protein concentrations were much lower in the serum samples from the exposure group than in the serum samples from the control group (*P* < 0.05). The creatinine concentrations were much higher for the exposure group (85.73 ± 14.16 *μ*mol/L) than the control group (79.55 ± 16.91 *μ*mol/L). The total bilirubin concentrations were also higher for the exposure group (14.11 ± 3.91 *μ*mol/L) than the control group (11.23 ± 5.08 *μ*mol/L). The concentrations of the other biochemical markers (total cholesterol, triglycerides, direct bilirubin, alanine aminotransferase, aspartate aminotransferase, blood urea nitrogen, and uric acid) for the exposure and control groups were not significantly different. The results are shown in Table 3.

### 3.7. ECG

The ECGs indicated that one person in the exposure group had coronary sinus rhythm, four had sinus arrhythmia, three had sinus arrhythmia, three had sinus bradycardia accompanied by arrhythmia, one had sinus arrhythmia and occasional atrial premature contraction, two had sinus arrhythmia with ST segment changes, four had sinus rhythm with ST segment changes, one had sinus rhythm with occasional ventricular premature contraction, two had sinus rhythm with left ventricular high voltage, two had sinus rhythm with clockwise transposition, two had sinus rhythm with incomplete right bundle branch block, and 46 had normal ECG. The ECGs indicated that three people in the control group had sinus arrhythmia, two had sinus bradycardia accompanied by arrhythmia, one had sinus arrhythmia with occasional premature atrial contraction, one had sinus tachycardia with T-wave changes, two had sinus arrhythmia with ST segment changes, four had sinus rhythm with ST segment changes, one had sinus rhythm with occasional premature ventricular contraction, one had sinus rhythm with preexcitation syndrome, two had sinus rhythm with a prolonged P–R interval, one had sinus rhythm with incomplete right bundle branch block, one had sinus rhythm with complete right bundle branch block, and 63 had normal ECG. The normal ECGs only included sinus rhythm and sinus rhythm with limb lead low voltage. Abnormal ECGs were found for 22 of the 68 people in the exposure group and 19 of the 82 people in the control group. The numbers of abnormal ECGs for the exposure and control groups were not significantly different (Table 4). The HR, PR interval, QRS interval, QT interval, QTc interval, RV5 amplitude, SV1 amplitude, and RV5+SV1 amplitude data for the exposure and control groups were compared. The HR was significantly lower for the exposure group (69.59 ± 9.63) than the control group (74.96 ± 7.87). The QT interval was significantly longer for the exposure group (median 387.00 ms (lower quartile 372.00 ms, upper quartile 394.50 ms)) than the control group (372.00 ms (lower quartile 360.00 ms, upper quartile 388.00 ms)). The QTc interval was also significantly longer for the exposure group (402.79 ± 15.30 ms) than the control group (393.60 ± 11.42 ms). The RV5, SV1, and RV5+SV1 amplitudes for the exposure and control groups were not significantly different (Table 5).

## 4. Discussion

Even though the ORs had laminar flow and scavenging systems, the antioxidant defense systems and vital organ functions of staff exposed to WAG in the long term could have been affected. However, no difference in DNA damage markers was found between the exposure and control groups. The time-weighted average sevoflurane concentrations in the ORs were lower than the Chinese limit of 2 ppm, but 28% of the air samples from the anesthesiologist work areas and 8% of the air samples from the OR nurse work areas had sevoflurane concentrations > 2 ppm. This indicated that OR staff may be exposed to high WAG concentrations at work.

Volatile inhaled anesthetics are widely used in ORs. However, anesthetic gas may escape into the OR while the anesthetic is being administered [11, 12]. Staff in the OR may therefore be exposed to WAG while working. There is much evidence suggesting that chronic exposure to WAG can increase oxidative stress [3, 5, 13] and cause genotoxicity [14] and carcinogenicity [15, 16]. In some large epidemiological investigations, medical staff exposed to WAG have been found to have increased risks of chronic diseases (e.g., cancer, liver dysfunction, and renal insufficiency), spontaneous abortion, and congenital malformation and to have low birth rates and high stillbirth rates [6–8]. However, most of these studies were performed decades ago. Recent changes in conditions, such as different anesthetic agents being used and OR ventilation equipment being installed, mean that exposure of OR staff to WAGs needs to be reassessed. We found relatively high levels of oxidative stress in OR staff exposed to WAG over a long period, similar to the results of previous studies [13]. Long-term exposure to WAGs can cause radical damage and the antioxidant defense system to become less effective. Long-term exposure to WAGs can decrease the SOD activity, GSH-px activity, trace element concentrations, and red blood cell count [2].

A series of stress responses can occur once DNA damage has occurred in a cell. These stress responses induce a signal cascade and even stop the cell cycle until the damage has been repaired. One of the main components of the signal cascade is H2AX, which can be phosphorylated when DNA double-strand breaks (DSBs) occur and then initiate the damage repair mechanism. H2AX plays an extremely important role in the DSB identification and repair process [17]. H2AX is a member of the histone H2A family. Phosphorylated H2AX recognizes DSBs, forms a focal point for DSBs, and participates in the recruitment of proteins to repair DSBs [18]. DSBs are closely related to *γ*H2AX production, possibly at a rate of one *γ*H2AX molecule per DSB [19]. *γ*H2AX is therefore considered to be a specific index for detecting DSBs in cells. The DNA damage marker concentrations in the exposure and control groups were not significantly different in our results. Similar results have been found in previous studies in which no significant changes were found in genotoxicity biomarkers in physicians using modern anesthesia workstations in ORs during medical residency programs [20, 21]. However, DNA damage caused by occupational exposure to WAGs remains controversial [22–24]. Costa et al. found increased risks of DNA damage and oxidative stress in young professionals and that more DNA damage and oxidative stress occurred after exposure to WAG for 22 months than after exposure to WAG for eight or 16 months [22]. El-Ebiary et al. and Chandrasekhar et al. found that exposure to WAG for >10 y caused DNA damage [25, 26]. Most published studies of the relationship between DNA damage and exposure to WAG were performed many years ago or in developing countries in which ORs did not contain laminar flow systems. The results could have been different in different previous studies because different sample sizes, anesthetic agents, air conditions, and WAG concentrations were used.

The blood lipid concentrations and liver and renal functions of the people exposed to WAG for a long time were compared. The total protein concentration in the peripheral blood and the total bilirubin and creatinine concentrations were significantly lower in the exposed group than the control group. The other biochemical indicators (total cholesterol, triglycerides, direct bilirubin, alanine aminotransferase, aspartate aminotransferase, blood urea nitrogen, and uric acid concentrations) in the exposed group and control group samples were not significantly different. In medical workers, exposure to low concentrations of anesthetic exhaust gas has been found to increase alanine aminotransferase, glutamyltransferase, and total bilirubin concentrations and lymphocyte and neutrophil counts [27]. Prokes [28] found mild changes in liver function in workers exposed to anesthetic exhaust. Caciari et al. [29] found that WAGs can affect liver and kidney functions. However, many factors (e.g., disease, fatigue, sleep, stress, alcohol, and medication) affect liver and kidney functions. More sensitive indicators and broader studies are required to determine whether WAGs affect blood lipid concentrations and liver and kidney functions.

The sevoflurane concentrations were, overall, lower than the Chinese workplace limit, but some OR staff (particularly anesthesiologists and anesthesia nurses, who work near their patients) could be exposed to sevoflurane concentrations higher than the Chinese workplace limit. Better laminar flow and ventilation systems therefore need to be installed in ORs, anesthesia delivery equipment needs to be inspected regularly for leaks, medical supervision needs to be maintained, and hazard awareness training is required. Actions to control WAG emissions (particularly installing and maintaining scavenging systems, using double masks, adequately managing airflow, and using closed or semiclosed low-flow anesthesia procedures) are required to decrease the exposure of OR staff to WAG as much as possible [30].

No significant difference was found between the rates of abnormal ECGs for the exposure and control groups. The HR was significantly lower for the exposed group than the control group, and the QT interval and QTc interval were significantly longer for the exposed group than the control group, although both were within the normal ranges. The PR intervals and RV5, SV1, and RV5+SV1 amplitudes for the exposure and control groups were not significantly different. Many factors (e.g., antiarrhythmic drugs, antibiotics, electrolyte disturbances, antipsychotic drugs, and sympathetic nerve excitement) affect the HR, QT interval, and QTc interval. Further studies are required to determine whether long-term exposure to sevoflurane at low concentrations changes the ECG parameters of anesthesiology staff.

The study had some limitations. First, the study was performed at a single site. Second, some biometric indicators (e.g., the concentrations of sevoflurane and its metabolite hexafluoroisopropanol in urine and blood) were not used to measure exposure to WAG. Third, only *γ*H2AX was used to measure DNA damage. *γ*H2AX reflects only DNA damage caused by recent exposure but not cumulative effects. More indicators of DNA damage should be assessed in future studies. Lastly, there could be many causes of changes in oxidative stress in the participants. Exposure to WAG may only be one of the causes of changes in oxidative stress. The contribution of exposure to WAG to changes in oxidative stress needs to be assessed in future studies.

## Figures and Tables

**Table 1:** Demographic characteristics of the exposure and control groups $\left(\bar{x}\pm s\right)$.

Group	Exposure group (*n* = 68)	Control group (*n* = 82)	*P* value
Sex			
Man	19	26	0.72
Woman	49	56	
Age	31.56 ± 5.64	29.54 ± 5.86	0.06
LOS	8.29 ± 5.15	7.37 ± 6.24	0.39
Smoking			
Yes	4	6	0.72
No	64	76	
Drinking			
Yes	6	9	0.78
No	62	73	
BMI	21.28 ± 2.37	21.17 ± 2.74	0.88

Notes. (1) Smoking was defined as smoking at least one cigarette per day for more than a year or having quit smoking for <1 y. (2) Drinking was defined as consuming alcohol at least once per week for more than half a year. (3) LOS: length of service. (4) BMI: body mass index.

**Table 2:** Complete blood count results for the exposure and control groups ($\left(\bar{x}\pm s\right)$ or median (lower quartile, upper quartile)).

Indexes	Exposure group (*n* = 68)	Control group (*n* = 82)	*P* value
HGB (g/L)	121.75 ± 12.89	133.62 ± 16.56	0.02
WBC (10^9^/L)	6.53 ± 1.60	7.04 ± 1.80	0.21
PLT (10^9^/L)	232.62 ± 53.15	228.55 ± 43.13	0.76
RBC (10^12^/L)	4.52 ± 0.51	4.70 ± 0.87	0.16
NEUT (10^9^/L)	3.77 ± 1.36	3.78 ± 1.58	0.99
LYM (10^9^/L)	2.31 ± 0.51	2.63 ± 0.82	0.04
EON (10^9^/L)	0.10 (0.06, 0.19)	0.11 (0.07, 0.25)	0.54
BASON (10^9^/L)	0.016 ± 0.009	0.018 ± 0.006	0.50

Notes. HGB: hemoglobin; RBC: red blood cell count; WBC: white blood cell count; PLT: blood platelet count; LYM: lymphocyte count; NEUT: neutrophil count; EON: eosinophil count; BASON: basophil cell count.

**Table 3:** Blood biochemical test results for the exposure and control groups ($\left(\bar{x}\pm s\right)$ or median (lower quartile, upper quartile)).

Indexes	Exposure group (*n* = 68)	Control group (*n* = 82)	*P* value
CHO (mmol/L)	4.70 ± 0.9	4.77 ± 0.95	0.77
TG (mmol/L)	0.83 (0.58, 1.23)	0.89 (0.65, 1.47)	0.39
TP (g/L)	70.65 ± 4.43	76.24 ± 5.61	0.03
T-BIL (*μ*mol/L)	14.11 ± 3.91	11.23 ± 5.08	0.03
D-BIL (*μ*mol/L)	4.53 ± 1.99	4.34 ± 2.05	0.73
ALT (u/L)	15.00 (12.00, 19.00)	14.50 (10.75, 27.25)	0.76
AST (u/L)	18.38 ± 5.42	20.91 ± 8.12	0.16
CRE (*μ*mol/L)	85.73 ± 14.16	79.55 ± 16.91	0.02
BUN (mmol/L)	5.25 ± 1.52	4.67 ± 1.29	0.32
UA (*μ*mol/L)	283.67 ± 74.26	296.74 ± 69.16	0.25

Notes. CHO: total cholesterol; TG: triglycerides; TP: total protein; T-BIL: total bilirubin; D-BIL: direct bilirubin; ALT: alanine aminotransferase; AST: aspartate aminotransferase; BUN: blood urea nitrogen; CRE: creatinine; UA: uric acid.

**Table 4:** Electrocardiogram results for the exposure and control groups.

ECG results	Exposure group (*n* = 68)	Control group (*n* = 82)	*χ* ^2^	*P* value
Normal	22	19	1.578	0.27
Abnormal	46	63		

**Table 5:** Electrocardiogram characteristics for the exposure and control groups ($\left(\bar{x}\pm s\right)$ or median (lower quartile, upper quartile)).

Indexes	Exposure group (*n* = 68)	Control group (*n* = 82)	*P* value
HR (bpm)	69.59 ± 9.63	74.96 ± 7.87	0.00
PR (ms)	149.9 ± 16.77	147.72 ± 17.91	0.38
QRS (ms)	89.45 ± 9.03	86.94 ± 8.19	0.13
QT (ms)	387.00 (372.00, 394.50)	372.00 (360.00, 388.00)	0.01
QTc(ms)	402.79 ± 15.30	393.60 ± 11.42	0.00
RV_5_(mV)	1.38 ± 0.45	1.35 ± 0.42	0.75
SV_1_(mV)	0.91 ± 0.42	0.89 ± 0.33	0.69
RV_5_+SV_1_(mV)	2.29 ± 0.70	2.25 ± 0.58	0.74

## Data Availability

All data used to support the findings of this study are available from the corresponding author upon request.
